# Strengthening health facilities for maternal and newborn care: experiences from rural eastern Uganda

**DOI:** 10.3402/gha.v8.24271

**Published:** 2015-03-31

**Authors:** Gertrude Namazzi, Peter Waiswa, Margaret Nakakeeto, Victoria K. Nakibuuka, Sarah Namutamba, Maria Najjemba, Ruth Namusaabi, Abner Tagoola, Grace Nakate, Judith Ajeani, Stefan Peterson, Romano N. Byaruhanga

**Affiliations:** 1 School of Public Health, College of Health Science, Makerere University, Kampala, Uganda; 2 INDEPTH, Iganga/Mayuge Health Demographic Surveillance Site, Kampala, Uganda; 3 Global Health, Department of Public Health Sciences, Karolinska Institutet, Stockholm, Sweden; 4 Kampala Children's Hospital, Kampala, Uganda; 5 St. Raphael of St. Francis Hospital, Nsambya, Kampala, Uganda; 6 School of Medicine, Uganda Martyrs University, Nkozi, Kampala, Uganda; 7 Ministry of Health, Kampala, Uganda; 8 International Maternal and Child Health Unit, Department of Women's and Children's Health, Uppsala University, Uppsala, Sweden

**Keywords:** health system strengthening, maternal care, newborn care, neonatal mortality, kangaroo mother care, Uganda

## Abstract

**Background:**

In Uganda maternal and neonatal mortality remains high due to a number of factors, including poor quality of care at health facilities.

**Objective:**

This paper describes the experience of building capacity for maternal and newborn care at a district hospital and lower-level health facilities in eastern Uganda within the existing system parameters and a robust community outreach programme.

**Design:**

This health system strengthening study, part of the Uganda Newborn Study (UNEST), aimed to increase frontline health worker capacity through district-led training, support supervision, and mentoring at one district hospital and 19 lower-level facilities. A once-off supply of essential medicines and equipment was provided to address immediate critical gaps. Health workers were empowered to requisition subsequent supplies through use of district resources. Minimal infrastructure adjustments were provided. Quantitative data collection was done within routine process monitoring and qualitative data were collected during support supervision visits. We use the World Health Organization Health System Building Blocks to describe the process of district-led health facility strengthening.

**Results:**

Seventy two per cent of eligible health workers were trained. The mean post-training knowledge score was 68% compared to 32% in the pre-training test, and 80% 1 year later. Health worker skills and competencies in care of high-risk babies improved following support supervision and mentoring. Health facility deliveries increased from 3,151 to 4,115 (a 30% increase) in 2 years. Of 547 preterm babies admitted to the newly introduced kangaroo mother care (KMC) unit, 85% were discharged alive to continue KMC at home. There was a non-significant declining trend for in-hospital neonatal deaths across the 2-year study period. While equipment levels remained high after initial improvement efforts, maintaining supply of even the most basic medications was a challenge, with less than 40% of health facilities reporting no stock-outs.

**Conclusion:**

Health system strengthening for care at birth and the newborn period is possible even in low-resource settings and can be associated with improved utilisation and outcomes. Through a participatory process with wide engagement, training, and improvements to support supervision and logistics, health workers were able to change behaviours and practices for maternal and newborn care. Local solutions are needed to ensure sustainability of medical commodities.

Neonatal conditions contribute approximately 10% to the global burden of disease, more than three times that of HIV (1). In Uganda this burden is doubled, with perinatal and maternal conditions contributing an estimated 20% of the overall burden of disease (2). While demand for facility deliveries seems to be increasing, many women still deliver in the community with the assistance of unskilled birth attendants such as traditional birth attendants, relatives, or even alone. After delivery, there are a number of traditional and socio-economic barriers to seeking care outside of the home for both healthy and sick newborns, as well as for mothers (3, 4). For those who do seek care at a health facility, substandard obstetric and neonatal care continues to be a factor in poor maternal and newborn outcomes (5, 6).

The importance of an integrated continuum of care from pre-pregnancy through childhood across levels of service delivery from household to hospital is well known (7). However, health system bottlenecks at all levels lead to low coverage of many priority interventions through poor coordination, weak infrastructure, shortage of trained and motivated health workers, low uptake of available capacity, and household resistance to recommended practices (8, 9). Within the World Health Organization Health System Building Blocks Framework, there are six components, namely health workforce, service delivery, information, supplies, financing, and leadership, which allow for the systematic identification of gaps within the system (10). This multidimensional approach helps identify synergistic effects of complementary interventions from facility level to district and up to national level, with careful monitoring and steering of dynamic and interrelated processes.

The Uganda Newborn Study (UNEST) was conceived to adapt a community care package for maternal and newborn health and evaluate its effect on maternal and newborn care outcomes in order to inform policy and scale-up in Uganda. When formative research revealed that poor-quality health facility care was the second leading reason for newborn deaths (4), it was determined that the intervention package needed to go beyond merely introducing a community-level cadre. Health facilities lacked infrastructure, equipment, drugs, supplies and protocols for newborn care, and the majority of health workers lacked knowledge and skills to care for vulnerable neonates (4). An increase in demand for services would not necessarily save lives without commensurate improvements in health facility quality.

Within Ugandan policy, one general hospital is supposed to serve approximately 500,000 people, while health sub-districts administer lower-level health facilities, including health centre (HC) levels II, III, and IV.; Level II HCs are small, outpatient-only units which can provide a first dose of antibiotics to sick newborns and referrals; level III HCs conduct births, manage newborn illness, and provide laboratory services; and Level IV HCs function as small hospitals, which should be equipped for emergency obstetric care. Private and faith-based organisations own 41% of hospitals and 22% of lower-level HCs. Private not-for-profit facilities receive government subsidies to expand care to rural areas (11).

Integration of the targeted interventions into a health system is difficult and complex, especially in a weak health system (12). Yet, strengthening health systems to deliver services equitably and efficiently is crucial for achieving improved maternal and newborn care. This paper describes the health systems strengthening process used to improve quality of care across 20 health facilities in rural eastern Uganda, and assesses its effect on the outcome of high-risk newborn babies. This paper is the fifth in a series on the UNEST.

## Methods

The study was nested within the UNEST randomised control trial whose details and results are described elsewhere (13, 14). Briefly, within UNEST, villages were either randomised to the intervention (a trained community health worker (CHW) making home visits to meet pregnant and newly delivered mothers) or the control (existing standard of care). UNEST was implemented in the Iganga/Mayuge Health and Demographic Surveillance Site with a population of around 70,000.

For the health systems strengthening component, 20 health facilities within the district were targeted from 2009 through 2011, including one hospital, one HC IV, six HC III, and twelve HC II, capturing both public and private health facilities. The health facilities catered to clients from both control and intervention areas, as well as outside of the study districts. For example, the catchment population of the hospital is approximately 1.5 million. The intervention was implemented together with district and Ministry of Health leadership to identify gaps in service delivery and to make changes which would improve the responsiveness of the health system to maternal and newborn complications.

We describe and analyse the health system changes according to the WHO Health System Building Blocks, including health workforce, service delivery, health information systems, equipment and supplies, with leadership and finance combined (10). Quantitative data were collected by mentorship teams from all health facilities from the routine health management information system (HMIS) on a quarterly basis. Baseline data were extracted for the 2 years prior to implementation. Quantitative data were obtained quarterly using a structured tool based on the national standards for newborn healthcare services (15) which was pretested prior to use.

An additional data capture form on service utilisation and care of the sick newborn babies and preterm babies using kangaroo mother care (KMC) was developed for use at the hospital. Key indicators collected included availability of basic essential medicines and equipment (e.g. newborn resuscitation equipment, neonatal weighing scale, thermometer, fetoscope, bulb syringe, blood pressure machine, stethoscope, delivery kit, steriliser, and light source); and availability of essential medicines (e.g. ampicillin, gentamycin, vitamin K, tetracycline eye ointment, Fansidar, oxytocin, and magnesium sulphate). Data on the number of deliveries, stillbirths, neonatal deaths, patients discharged, as well as health worker training and turnover were collected. In instances where routine data were incomplete, stock cards were used to complete the section on medicines. Data were entered into Microsoft Excel spreadsheets and checked for completeness, then exported to Epidata version 3.1 for descriptive analysis.

As part of the quarterly process monitoring, qualitative data were also collected. The study team documented implementation experiences and contextual factors and events (e.g. new facility management, major donor input to a facility or community) that might influence study outcomes. Field notes and reports from the support supervision and mentorship visits were reviewed on a quarterly basis. These activities were the basis for information on performance of service providers.

### Health workforce

Health workers at the targeted facilities were identified and their level of formal training documented. With involvement of facility administration and district leadership, frontline workers attended in-service refresher training in obstetric and newborn care, modified for the level of service delivery. Four training sessions were conducted between July 2009 and June 2010. Each training session lasted 6 days and used an integrated training maternal and newborn manual developed by the study team, reviewed by members of national midwifery, obstetric and paediatric professional associations, and endorsed by local stakeholders and the Ministry of Health. The training package addressed the major causes of maternal and newborn deaths and morbidity in Uganda. Modules included goal-oriented antenatal care, managing maternal complications, infection prevention, managing normal labour and partograph use, neonatal resuscitation, care of the sick newborn, and extra care for the small baby using KMC. Knowledge tests were given before and after training, as well as after 1 year of implementation. In addition, training in maternal and perinatal mortality auditing was provided according to Ministry of Health guidelines (16).

### Service delivery

In order to improve service delivery, the organisation of wards and patient flow were examined for critical bottlenecks. Interventions involved the redesigning and reorganisation of space to cater for labour management and newborn care. Screens were introduced for privacy in the labour ward. In the hospital, space was specifically designated for KMC by screening off two beds and placing posters and job aids on the walls. Later, with district buy-in, a modest special care neonatal unit was built adjacent to the labour and delivery ward to care for high-risk newborns.

Support supervision and mentoring was also a key component of improving service delivery. Teams of national-level and district-based health workers carried out quarterly outreach visits to facilities that were conducting deliveries. The co-opted mentoring team comprised a paediatrician, an obstetrician, a midwife, and district technical officers. The supervision visits included assessment of skills, support for problem solving with frontline staff to identify gaps and solutions, and development and review of health facility work plans. In initial outreach visits, an assessment of the physical layout of the sites was done with recommendations for reorganisation, if needed. Maternal and perinatal death review committees were established with the expectation of biweekly meetings. The minutes were reviewed during supervision visits. Newly introduced interventions, such as neonatal resuscitation using bag and mask, KMC, and treatment of neonatal sepsis, were given particular attention during mentorship visits.

### Equipment and supplies

Sensitisation meetings were held with health administration officials at different levels to highlight maternal and newborn care needs and the importance of timely procurement and dispatch of supplies. Health facility in-charges and district health teams were engaged to ensure future supplies are provided for through the routine channels. A once-off supply of essential medicines, medical supplies, and equipment was provided as a catalyst to address immediate gaps. Supplies included delivery beds, Ambu bags, newborn-size masks, bulb syringes, nasal prongs, cannulas, and oxygen concentrators. Partographs and standard management protocols and guidelines were printed and distributed targeting major causes and solutions of neonatal morbidity and mortality.

### Health information

The status of the patient charts and registers was assessed at baseline. File folders for inpatient care in maternity and paediatric wards approved by the Ministry of Health were reintroduced to standardise record-keeping and to facilitate data availability for the audit sessions. Where lacking, registers and summary sheets were printed and provided. At the hospital, new registers were developed and provided for the KMC unit, the neonatal special care unit, and the neonatal room on the paediatric ward. Forms for maternal death notification and maternal and perinatal mortality audit were also provided. During support supervision, health workers and records staff were consulted about the completeness and accuracy of the information collected. The HMIS data were extracted on a quarterly basis by the support supervision team.

### Leadership/governance and finance

Sensitisation of district leaders, CHWs, and various stakeholders on the importance of maternal and newborn health was an early intervention milestone, with the assumption that local buy-in was crucial. Managers at health units were encouraged to prioritise procurement of basic supplies for obstetric and newborn care, and were given strategies to avoid stock-outs, support for annual budgeting, and training on quality improvement. The identification and development of local champions was a key strategy. Individuals were identified during early trainings and later involved in the co-opted mentorship teams, learning visits, trainings, reviews, and dissemination activities.

## Results

Amongst the facilities assessed, all offered antenatal care, well-baby checkups, and served as a point of contact for sick newborns. Fourteen of the facilities (70%) conducted deliveries. Although current Ugandan policy only mandates delivery services at level III HCs and above, it was found that half of the level II HCs were also conducting deliveries. Only the hospital had facilities for admission and treatment with antibiotics for sick neonates.

**Fig. 1:**
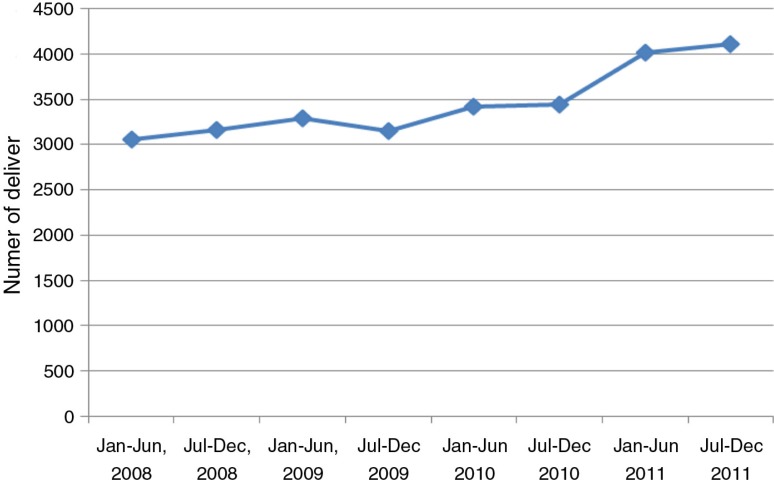
Health facility deliveries from Iganga/Mayuge Demographic Surveillance Site.

During the implementation period, there was an increase in the number of deliveries conducted at health facilities within the study period, from 3,151 at the beginning of the intervention in the second half of 2009, to about 4,115 deliveries (an increase of 30%) at the end of the study (Fig. 1). Despite this increase, there was no change in the proportion of births resulting in caesarean section, which was 12% at the beginning of implementation in 2009 and 13% by the end of implementation. The rate of preterm birth was 8% in deliveries occurring in health units. The number of sick neonates from the community admitted to the neonatal unit also increased. A total of 249 sick newborn babies were admitted to the paediatric neonatal unit during the study period. The in-hospital neonatal mortality rate amongst admitted sick neonates declined from 17% in the first quarter to 9% in the last quarter, although the trend was non-significant.

### Health workforce

**Fig. 2:**
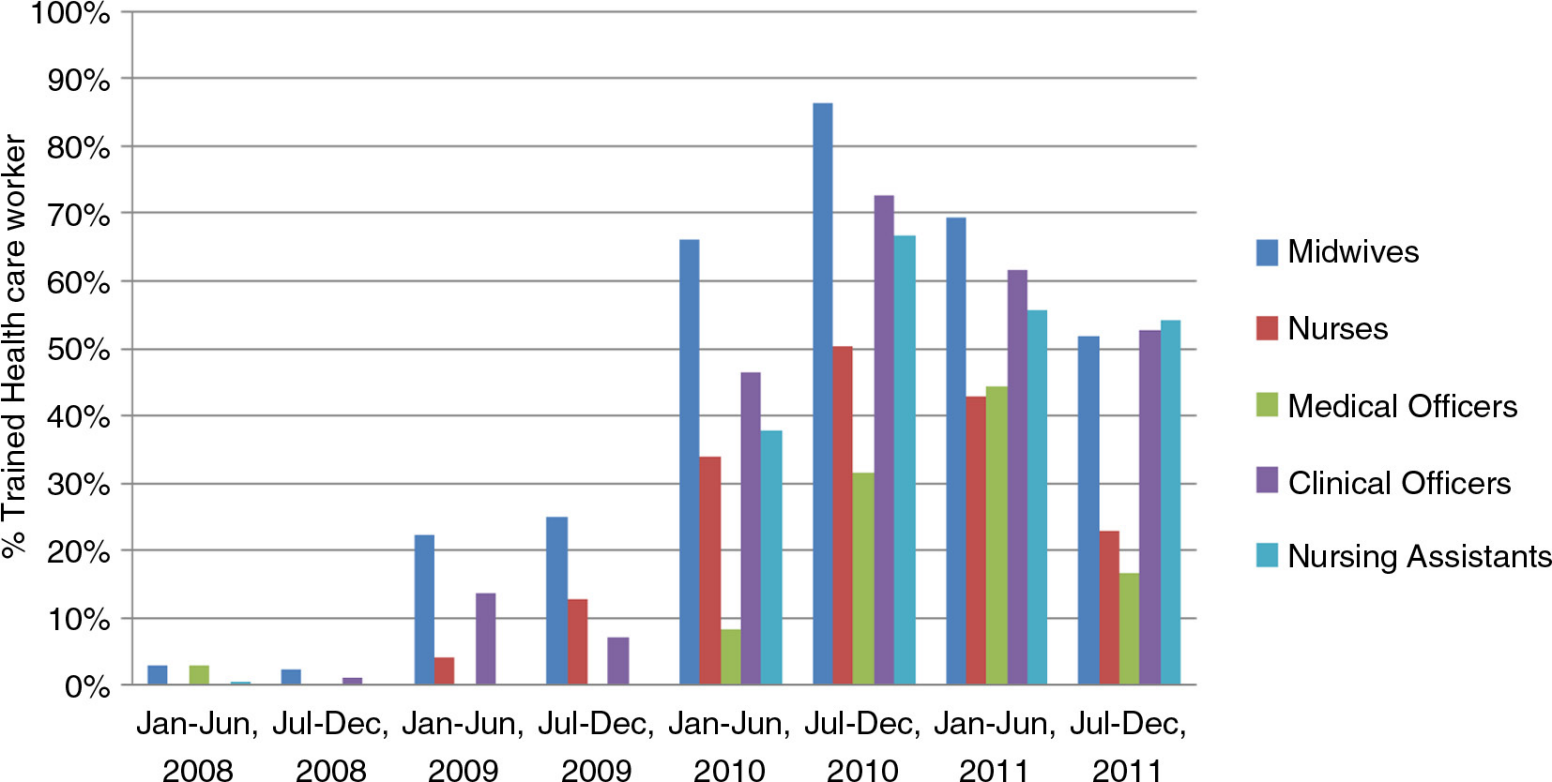
Proportion of health workers trained in maternal and newborn care by cadre.

Over the 2-year implementation period, four in-service training sessions were conducted. Overall, 72% (105/146) of targeted health providers were trained (Table 1). The majority of trainees were midwives, nurses, and nursing assistants. Ten clinical officers and one medical officer also received training (Fig. 2). A higher proportion of the staff at lower-level health units was trained, although absolute numbers were higher for hospitals.

**Table 1:** Distribution of health facilities by level and eligible health workers trained

Health facility level	Number of health facilities	Total number of health workers	Health workers trained (%)
HC II	12	33	25 (76)
HC III	6	49	34 (69)
HC IV	1	14	12 (86)
Hospital	1	50	34 (68)
Total	20	146	105 (72)

HC *=* Health centre.

Results from pre-training and post-training tests demonstrated improvement in knowledge of maternal and newborn care. The mean pre-training score was 32% compared to a post-training score of 68%. After 1 year of implementation, the mean score was 80%. While skill retention was not systematically assessed, this was documented in supervision notes. Supervision reports also revealed that health workers, midwives in particular, gained confidence and skills in inserting intravenous cannulas for medicines and dextrose, as well as nasogastric tubes for feeding preterm babies expressed breast milk, and for conducting neonatal resuscitation. In terms of identifying and supporting champions, most facilities had specific staff that took up more responsibility and had more enthusiasm for the relatively novel area of newborn care, and used their time to mobilise and train others.

### Service delivery

Infrastructural improvements, particularly the creation of a KMC unit, transformed service delivery (Box 1). While at baseline there were no additional services available for small babies at the hospital or HCs, by the end of the study, 547 preterm babies had been cared for in a KMC unit. Of those admitted to the hospital's KMC unit, 85% were discharged alive, with less than 2% of admissions requiring referral to higher-level care. Three-quarters of mother–baby pairs completed KMC follow-up to ‘graduate’ from the service. A substantial proportion (13%) of women admitted to KMC self-discharged against medical advice. Attempts were made to follow-up these cases in the community, and where possible they were linked to a lower-level health facility for continued care. Other infrastructural adjustments, such as the partitioning of the paediatric ward to allocate space for newborns, resulted in neonates being seen as needing special care apart from maternity and older child services. Differences between public and private facilities were observed, with the practice of essential newborn care services slightly higher in public facilities (17).

Box 1 **Twins benefit from newly introduced kangaroo mother care (KMC) practice**

When KMC was first being introduced at the hospital, during a supervision visit, the mentorship team found a woman admitted to the maternity ward who had delivered premature twins by caesarean section 6 days prior. The babies were weak, hypothermic, and were being fed glucose water. Their mother had painful, engorged breasts and remained weak following surgery.

The supervision team counselled the health workers to admit the mother and babies to the KMC unit. The mother was supported to express breast milk while nasogastric tubes were passed for feeding the babies. The health workers were instructed on how to calculate feeds and do daily routine monitoring, including weighing. The grandmother and the mother of the twins were counselled on carrying the babies skin-to-skin while the mother regained strength and could take over the skin-to-skin care. The mother and twins were discharged after 3 weeks, and came back for weekly reviews.

Treatment protocols on admission, feeding, monitoring, and discharge were developed and posted on the walls of the KMC unit. A motivated midwife was identified to champion KMC amongst staff and mothers and to serve as a point person for the mentorship team. While this single-champion strategy was successful as a short-term solution, high turnover amongst staff means that a broader leadership or steering team for KMC is beneficial.

Supervision visits and learning opportunities were well utilised. Based on competencies observed during supervision visits, staffing adjustments were made by the district health team. Supervision visits lasted 3 days each, with half a day spent at each lower-level HC and 1 full day at the hospital. Exchange visits were provided for hospital staff at the national referral hospital and a general hospital in the capital city of Kampala for additional mentorship. Despite widespread national and local support for the mortality review process, this was not well taken up by facility staff and administration, citing competing demands and excessive workload. Fear of blame for specific failures in care might also have been a reason for discontinuation.

### Equipment and supplies

At the level IV HC and hospital, less than half of the essential equipment was available and/or functional at baseline, with level II and III HCs functioning better for supplies, with 70% being available. Following the initial procurement and distribution of supplies to health facilities, these levels were maintained and even increased in the level IV HC and hospital. Still, only 80% of level II and III HCs had the basic equipment necessary by the end of the implementation period. Due to increasing demand for services and despite support for procurement, stock-outs of essential medicines were common throughout the implementation period and remained below 40%. This was more pronounced in the level IV HC and hospital compared to the lower-level health facilities, both due to increasing demand and budgetary constraints of the ‘pull’ procurement system.

### Health information

The availability and quality of health information improved dramatically over the course of the implementation. Whereas at baseline there were very few indicators captured within the routine system for neonates, outcome data became readily available and were requested by administrators and district leaders through the HMIS. Partograph use and completeness, documentation of weight, feeding, and treatments received all increased. The completeness and accuracy of inpatient and outpatient register documentation improved along with individual patient charts, although these were dependent on continued availability of stationery after the initial seed stock was consumed.

### Leadership/governance and finance

Creating linkages among the health workers, community leaders, and district officials was an important part of ensuring continuity of care from the provider side. Health workers met with CHWs on a monthly basis at the health facility, and once a month they accompanied CHWs on home visits. Through community meetings, challenges at community level and health facility were discussed and raised with leadership, for example, the lack of housing available for health facility staff (Box 2). Managers were key drivers of all of the quality improvement processes employed. However, some of the major health systems bottlenecks (e.g. availability of finances for maintaining the supply of essential medicines) were outside of the control of local managers.

Box 2 **Ensuring availability of essential equipment and supplies: a health centre (HC) III case study**

According to national policy, level III HCs should be able to perform normal deliveries. However, at baseline, it was identified that one HC III had two midwives on its staff but was lacking all basic equipment, including a delivery bed, clean delivery kits, and a surface for resuscitation. Most of the women in the sub-county were delivering at home or at the home of a traditional birth attendant due to the long distance to the next nearest facility.

Upon initiation of UNEST, the HC III received an initial seed stock of supplies, equipment, and essential medicines. Through mentorship visits, the maternity ward was reorganised for efficiency. Staff received support for using equipment and documenting care. While the number of births in the facility steadily increased, the ability to provide around-the-clock services was constrained given the lack of accommodation for midwives at or near the facility. Upon discussion with sub-county leaders and the district health team, it was agreed that housing would be donated by the community and constructed and provided onsite.

## Discussion

The health systems strengthening efforts in this study reflect the limited material input possible within the existing district structure. The selected interventions were identified together with the Ministry of Health, district health team, and local experts, and were within the confines of the Uganda National Minimum Health Service Package. The number of deliveries with a trained, equipped birth attendant increased, with substantial improvements in the care provided to sick and preterm babies, particularly in public sector facilities. A strategy involving simple structural improvements, in-service training opportunities, effective team-based mentoring, and improved documentation has the potential to strengthen the capacity of the providers to care for vulnerable newborn babies. However, the shortage of essential drugs and supplies remains a major bottleneck in the system.

Simple partitioning within the delivery, postnatal and paediatric wards to provide a designated space for newborns likely contributed to improved awareness of newborn care in the community. Interviews with mothers and health providers demonstrated increased confidence in the services they were receiving and providing (14). The rate of self-discharge amongst women admitted to KMC possibly reflects the additional cost to families (e.g. meals, care for children at home) for inpatient care and also lack of exposure to KMC as a treatment regimen. Mechanisms to alleviate this burden and increase demand for KMC services should be explored. Given that KMC was only introduced at the hospital, there is an opportunity for step-down services to be rolled out to lower-level health facilities, and allowing services to be provided closer to home for many families.

Based on feedback following trainings, the in-service training was well accepted. The increase in provider knowledge 1 year after training reflects the importance of mentorship visits and support supervision. Increased frequency of *in situ* training is likely to further improve health worker skills, as demonstrated in Tanzania (18). Recent updates to the national nursing and midwifery training curriculum to cover aspects of newborn care, including KMC, will ensure that future staff have additional exposure to maternal and newborn complications as well as caring for sick and small babies. Ongoing support supervision and mentorship in addition to learning opportunities supported the retention of knowledge and skills of previously trained providers. The development of champions within the hospital and HCs was critical, particularly to support the newly introduced KMC service.

The equipment and medicines provided reflect only the most basic supplies necessary to provide maternal and newborn care. Yet, after the initial catalytic supply, the availability of medicines was erratic. While equipment levels were higher throughout the study period, there were also issues around maintenance and replacement. While administrative officials were encouraged to prioritise procurement, more effort is needed to improve budgeting and governance structures and to engage with the central medical stores to ensure that these are available. With an increasing volume of births in an area of high fertility, accurate planning for the quantity of necessary commodities is essential. Factors such as the availability of accommodation for service providers and the lack of ambulances and other emergency transport services were identified, but are largely beyond the ability of the study to address in the short term.

There are several limitations to this study. First, although data were compiled by study researchers, they were collected within routine systems. Improving data collection was one of the interventions targeted through supervision visits, which may present bias. In addition, the study did not assess the quality of care provided, nor were health worker skills observed and evaluated systematically. While health facility strengthening was introduced alongside the randomised community-based package, it is not likely that the increase in awareness of and demand for services was solely related to the community intervention, given that the catchment area for the facilities, particularly the hospital, is well beyond the implementation districts. The reasons for the increase in utilisation of services are multifactorial, but it is possible that the intervention contributed to this increase as well as to the overall quality of services provided to families.

Finally, given that there was little prior experience with maternal and newborn care services, the quality improvement interventions were introduced stepwise, which may have limited their effect, because there was a limited time period for the new services to take hold and be implemented together. An independent examination of mortality effect and impact on neonatal outcomes would be beneficial to supplement routine data and highlight potential gaps in measurement.

## Conclusion

Improving awareness of and demand for maternal and newborn care services at community level necessitates engagement with health facilities in order to ensure services are available and of sufficient quality. Even amongst higher-level health facilities, availability and quality of maternal and newborn care was low or non-existent. Basic health system strengthening was feasible in this low-resource setting, mostly within existing resources. Alignment with the Ugandan minimum health service package and the national standards for newborn health care demonstrates the potential for replication in other districts. Local leadership support, particularly engaging key champions and ensuring buy-in from frontline health providers, is required from the outset. However, addressing quality of care bottlenecks is a significant challenge and further innovative solutions are needed for resource constrained settings in order to save the lives of mothers and babies and help them thrive.

## References

[1] Lawn JE, Blencowe H, Darmstadt GL, Bhutta ZA (2013). Beyond newborn survival: the world you are born into determines your risk of disability-free survival. Pediatr Res.

[2] MoH (2005). Health sector strategic plan II 2005/06–2009/10.

[3] Kallander K, Hildenwall H, Waiswa P, Galiwango E, Peterson S, Pariyo G (2008). Delayed care seeking for fatal pneumonia in children aged under five years in Uganda: a case-series study. Bull World Health Organ.

[4] Waiswa P, Kallander K, Peterson S, Tomson G, Pariyo GW (2010). Using the three delays model to understand why newborn babies die in eastern Uganda. Trop Med Int Health.

[5] Mbonye AK, Asimwe JB, Kabarangira J, Nanda G, Orinda V (2007). Emergency obstetric care as the priority intervention to reduce maternal mortality in Uganda. Int J Gynaecol Obstet.

[6] Waiswa P, Kemigisa M, Kiguli J, Naikoba S, Pariyo GW, Peterson S (2008). Acceptability of evidence-based neonatal care practices in rural Uganda – implications for programming. BMC Pregnancy Childbirth.

[7] Kerber KJ, de Graft-Johnson JE, Bhutta ZA, Okong P, Starrs A, Lawn JE (2007). Continuum of care for maternal, newborn, and child health: from slogan to service delivery. Lancet.

[8] Okuonzi SA (2004). Learning from failed health reform in Uganda. BMJ.

[9] MoH (2008). Situation analysis of newborn health in Uganda. Current status and opportunities to improve care and survival.

[10] WHO (2007). Everybody's business – strengthening health systems to improve health outcomes: WHO's framework for action.

[11] Lundberg M, Amin S, Das J, Goldstein M (2008). Client satisfaction and the perceived quality of primary health care in Uganda. *Are You Being Served? New Tools for Measuring Service Delivery*.

[12] Atun R, de Jongh T, Secci F, Ohiri K, Adeyi O (2010). A systematic review of the evidence on integration of targeted health interventions into health systems. Health Policy Plan.

[13] Waiswa P, Peterson SS, Namazzi G, Ekirapa EK, Naikoba S, Byaruhanga R (2012). The Uganda Newborn Study (UNEST): an effectiveness study on improving newborn health and survival in rural Uganda through a community-based intervention linked to health facilities – study protocol for a cluster randomized controlled trial. Trials.

[14] Waiswa P, Pariyo G, Kallander K, Akuze J, Namazzi G, Ekirapa-Kiracho E (2014). Effect of the Uganda Newborn Study on care-seeking and care practices: a cluster-randomised controlled trial. Glob Health Action.

[15] Ministry of Health (2010). Newborn implementation framework: standards for newborn health care services.

[16] MoH (2010). Maternal and perinatal death review guidelines.

[17] Waiswa P, Akuze J, Peterson S, Kerber K, Tetui M, Forsberg BC (2014). Differences in essential newborn care at birth between private and public health facilities in eastern Uganda. Glob Health Action.

[18] Mduma E, Ersdal HL, Svensen E, Perlman J (2014). Low-dose high-frequency simulation training reduces early neonatal mortality. International Meeting on Simulation in Healthcare (IMSH).

